# Iterative multi-channel radio frequency pulse calibration with improving B_1_ field uniformity in high field MRI

**DOI:** 10.1186/s12938-015-0010-z

**Published:** 2015-02-21

**Authors:** Daniel Hernandez, Min Hyoung Cho, Soo Yeol Lee

**Affiliations:** Department of Biomedical Engineering, Kyung Hee University, Yongin-si, Gyeonggi-do 446-701 Korea

**Keywords:** RF pulse calibration, MRI, B_1_ uniformity, B_1_ mapping, B_1_ shimming

## Abstract

**Background:**

In high field MRI capable of multi-channel radio frequency (RF) transmission, **B**_1_ shimming is a time-consuming job because conventional **B**_1_ shimming techniques require **B**_1_ mapping for each channel. After acquiring the complex-numbered **B**_1_ field maps, the optimal amplitude and phase of the driving RF pulse are determined for each channel to maximize the **B**_1_ field uniformity in conventional **B**_1_ shimming. However, time-consuming **B**_1_ shimming procedures at the pre-scan may not be tolerated in the clinical imaging in which patient throughput is one of the important factors.

**Methods:**

To avoid the time-consuming **B**_1_ mapping, the first spin echo and the stimulated echo were repeatedly acquired in the slice-selective stimulated echo sequence without imaging gradients. A cost function of the amplitudes and phases of the driving RF pulse for every channel was defined in a way that the ratio between the spin echo and stimulated echo amplitudes rapidly converged to √ 2. The amplitude and phase of the driving RF pulse were iteratively modified over the repeating RF pulse sequence so that the cost function was minimized.

**Results:**

From the finite-difference-time-domain (FDTD) electromagnetic field simulations with a human body model placed in a birdcage coil operating at 3 T, it was observed that the RF pulse calibration with iterative cost function minimization can give improvement of B_1_ field uniformity as well as flip-angle calibration. The experiments at 3 T also showed improvement of RF field uniformity in the phantom imaging studies.

**Conclusions:**

Since the proposed RF pulse calibration is not based on **B**_1_ mapping, the RF pulse calibration time could be much shorter than the **B**_1_-mapping based methods. The proposed method is expected to be a practical substitute for the **B**_1_-mapping-based **B**_1_ shimming methods when long pre-scan time is not tolerable.

## Background

Radio frequency (RF) pulse calibration, also called flip angle calibration, is one of the calibration procedures which are performed in the pre-scan of MRI. The main objective of RF pulse calibration is to find the RF pulse amplitude that makes the desired flip angle, usually 90 degrees, over the region of interest. The RF pulse calibration time is usually negligible if the RF magnetic field is uniform over the imaging region. However, if the RF magnetic field, often called **B**_**1**_ field, is highly inhomogeneous in the human body as in high field MRI, the RF pulse calibration may become troublesome since the flip angle is also inhomogeneous over the imaging region in the human body. **B**_1_ shimming is the technique to improve the RF field homogeneity in high field MRI. To make **B**_1_ shimming, the MRI system must be equipped with a multi-channel RF transmission system to drive a multi-channel transmit coil [1,2]. By driving the multi-channel transmit coil with optimal amplitude and phase, the RF field homogeneity can be greatly improved over the imaging region in high field MRI [3-5]. However, B_1_ shimming requires **B**_1_ field mapping for each channel of the multi-channel transmit coil to determine the optimal amplitude and phase of the RF pulse at each channel. Although many fast **B**_1_ mapping techniques have been developed [6-16], the extra scan time for **B**_1_ mapping for every channel would not be acceptable in the clinical pre-scan.

We propose a fast RF pulse calibration technique that does not require time-consuming **B**_1_ mapping to drive the multi-channel transmit coil with RF pulses of optimal amplitude and phase. For the RF pulse calibration, we use the stimulated echo pulse sequence consisting of three RF pulses of the same amplitude without applying any imaging gradients. We repeat the RF pulse sequence with changing the amplitude and phase of the driving RF voltage at each channel. The optimal amplitude and phase are found by minimizing the cost function, defined on the magnitudes of the first spin echo and the stimulated echo, during the idle time between the RF pulse sequence. With the cost function minimization, the RF pulse calibration can give us improvement of the RF field homogeneity as well as the flip angle calibration. We present finite-difference-time-domain (FDTD) simulation results along with the experimental results performed at 3 T MRI.

## Methods

### B_1_ field formed by multi-channel transmission

**B**_1_ field in MRI is the circularly polarized magnetic field rotating with Larmor frequency in the same direction as the spin precession. If one channel of the multi-channel RF coil is independent on the other channels, the composite RF magnetic field becomes the sum of the RF magnetic fields generated by each channel:

1 $$ {\mathbf{B}}_1^C\left(x,y\right)={\displaystyle \sum_{i=1}^N{\mathbf{a}}_i{\mathbf{B}}_1^i}\left(x,y\right) $$

in which ${\mathbf{B}}_1^i$ is the complex-numbered circularly polarized component of the RF magnetic field generated by the *i*-th channel when driven by the unit voltage, and **a**_*i*_ is the complex-numbered driving voltage at the *i*-th channel. Since ${\mathbf{B}}_1^i$ has spatial distribution over (*x*, *y*) and is complex numbered, the composite field ${\mathbf{B}}_1^C$ may be manipulated to have uniform spatial distribution by changing **a**_*i*_, so called **B**_1_ shimming.

If there are only two channels for the RF transmission, as in the case of two-port birdcage coil in this study, the composite RF magnetic field is written as,

2 $$ {\mathbf{B}}_1^C\left(x,y\right)={\mathbf{a}}_1{\mathbf{B}}_1^1\left(x,y\right)+{\mathbf{a}}_2{\mathbf{B}}_1^2\left(x,y\right)={a}_1{e}^{j{\theta}_1}{\mathbf{B}}_1^1\left(x,y\right)+{a}_2{e}^{j{\theta}_2}{\mathbf{B}}_1^2\left(x,y\right) $$

in which ${\mathbf{a}}_1={a}_1{e}^{j{\theta}_1},\ {\mathbf{a}}_2={a}_2{e}^{j{\theta}_2}$ are the complex-numbered driving voltages for port 1 and 2, respectively. In an MRI system with multi-channel transmission capacity, the magnitudes of the driving voltages, *a*_1_ and *a*_2_, and the phases of the driving voltages, *θ*_1_ and *θ*_2_, can be controlled independently at the stage of RF pulse generation at the spectrometer. Therefore, the composite RF field can be considered as a function of the control parameters at the spectrometer as well as the spatial coordinates, ${\mathbf{B}}_1^C\left({a}_1,{a}_2,{\theta}_1,{\theta}_2,x,y\right).$

### RF pulse calibration sequence

**Figure 1:**
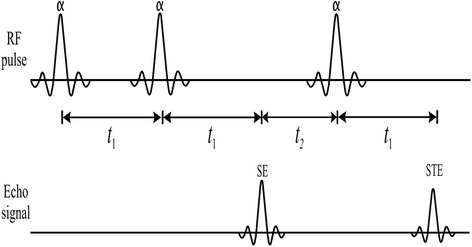
**The RF pulse calibration sequence.** Three identical RF pulses are applied in sequence at each channel of the transmit coil, and the first spin echo (SE) and the stimulated echo (STE) are acquired to determine the magnitudes and phases of the RF pulses to be applied at the next RF pulse sequence.

When the composite RF field is applied to flip the longitudinal magnetization onto the transversal plane, the flip angle distribution is given by,

3 $$ \alpha \left(x,y\right)=\gamma \left|{\mathbf{B}}_1^C\left({a}_1,{a}_2,{\theta}_1,{\theta}_2,x,y\right)\right|{\displaystyle {\int}_0^{T_p}A(t)dt} $$

in which *A*(*t*) is the RF pulse waveform and γ is the gyromagnetic ratio. Since the flip angle distribution is directly related with the **B**_1_ field distribution, the flip angle shimming is equivalent to the **B**_1_ shimming. The pulse sequence shown in Figure 1 is used for the RF pulse calibration in this study. The pulse sequence consists of three slice-selective RF pulses of the same magnitude. With this pulse sequence, two echo signals, the first spin echo (SE) and the stimulated echo (STE) are acquired for the RF pulse calibration. The other three echoes appearing after the third RF pulse are suppressed during the data acquisition or moved away from the data acquisition window by manipulating the timing of the slice-selective gradient [17]. If the flip angle *α* is uniform over the slice, the magnitudes of the first spin echo and the stimulated echo are given by [17],

4 $$ SE={M}_0 \sin \alpha { \sin}^2\frac{\alpha }{2} $$

5 $$ STE=0.5{M}_0{ \sin}^3\alpha $$

where *M*_0_ is the equilibrium magnetization of the slice.

### Iterative adjustment of the shimming parameters

In **B**_1_ shimming, the optimal magnitudes and phases of the driving RF pulses are determined from the **B**_1_ maps acquired beforehand. Rather than adopting time-consuming **B**_1_ mapping procedure, iterative adjustment of the shimming parameters, *a*_1_, *a*_2_, *θ*_1_, *θ*_2_ in this study, is employed. A cost function is defined on *SE* and *STE*, and optimal shimming parameters are found by minimizing the cost function of *SE* and *STE*,

6 $$ {\left({a}_1,{a}_2,{\theta}_1,{\theta}_2\right)}_{\mathrm{opt}}=\underset{a_1,{a}_2,{\theta}_1,{\theta}_2}{\mathrm{argmin}}\mathrm{C}\left(SE,STE\right). $$

The cost function is defined as,

7 $$ \mathrm{C}\left(SE,STE\right)=\left|{ \cos}^{-1}\left(\frac{ST{E}^2}{S{E}^2}-1\right)\right|. $$

**Figure 2:**
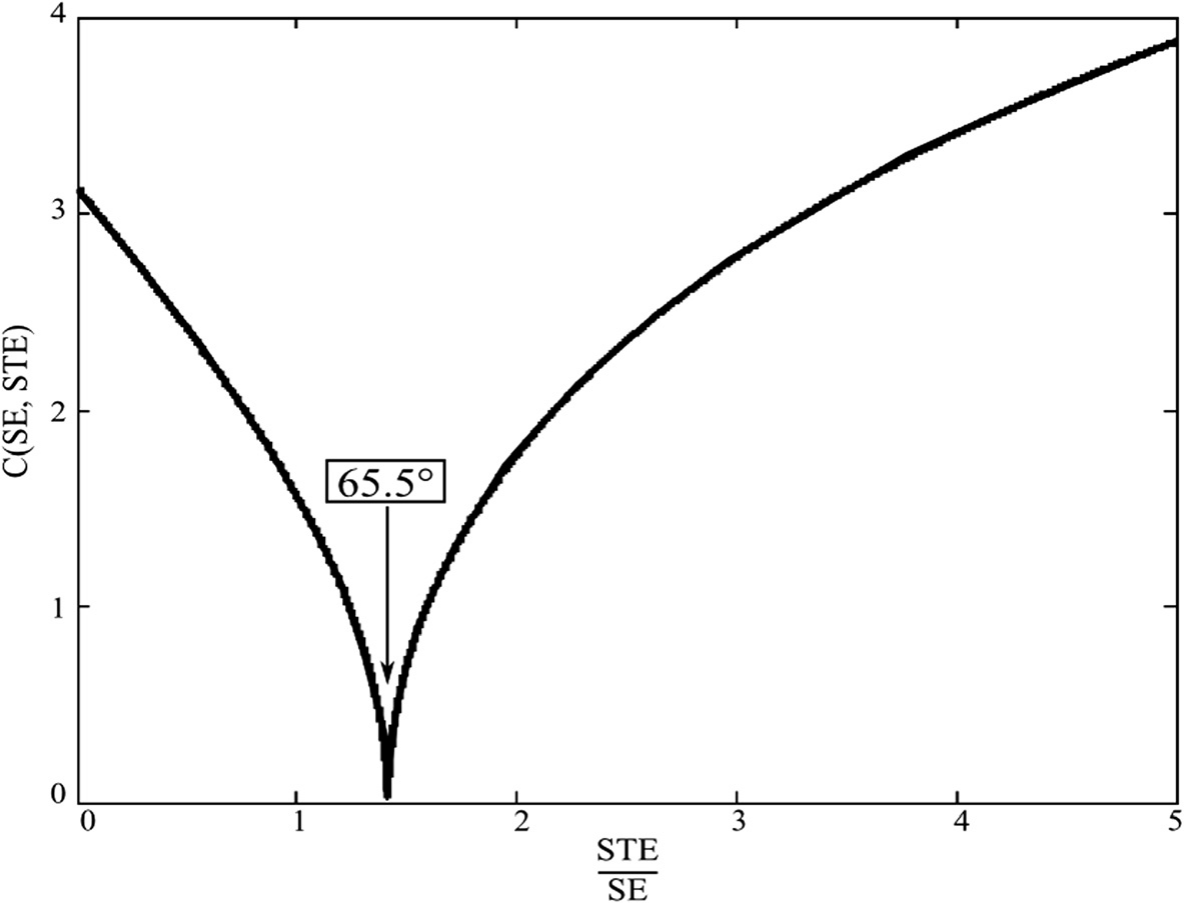
**The cost function value with respect to the ratio of STE to SE.** The cost function value becomes 0 radians at the flip angle of 65.5 degrees.

The cost function has the minimum value of 0 radians when the ratio of *STE* to *SE* is as below,

8 $$ \frac{STE}{SE}=\frac{0.5{ \sin}^2\alpha }{{ \sin}^2\frac{\alpha }{2}}=\sqrt{2} $$

which gives *α* value of 65.53 degrees. This means that if the shimming parameters are adjusted to make the ratio of $\sqrt{2},$ the flip angle becomes 65.53 degrees if the RF field is homogeneous. Figure 2 shows the cost function with respect to the ratio. The sharp dip around 65.53 degrees implies that the cost function converges to the minimum point rapidly with possible robustness to the perturbation like the noise or system instability.

If the RF field is inhomogeneous over the imaging region in which the spins are distributed by ρ(*x*,*y*), the first spin echo and the stimulated echo are given by,

9 $$ \begin{array}{l}\mathbf{S}\mathbf{E}={\displaystyle \iint \rho \left(x,y\right) \sin \alpha \left(x,y\right){ \sin}^2\frac{\alpha \left(x,y\right)}{2}}{e}^{j\varphi \left(x,y\right)} dxdy\\ {}\mathbf{S}\mathbf{T}\mathbf{E}={\displaystyle \iint 0.5\rho \left(x,y\right){ \sin}^3\alpha}\left(x,y\right){e}^{j\varphi \left(x,y\right)} dxdy\end{array} $$

in which ∅(x,y) is the transceiver phase of the MRI system. The transceiver phase is determined by the sum of the transmit field phase of the transmitting coil and the receive field phase of the receiving coil. Since both the flip angle and the transceiver phase are inhomogeneous over the imaging region, the ratio of $\sqrt{2}$ between *SE* = |**SE**| and *STE* = |**STE**| does not guarantee either the nominal flip angle of 65.53 degrees or uniform formation of the **B**_1_ field. However, the narrow dip of the cost function shown in Figure 2 suggests that the optimization of the cost function with respect to the shimming parameters may result in uniform **B**_1_ field.

**Figure 3:**
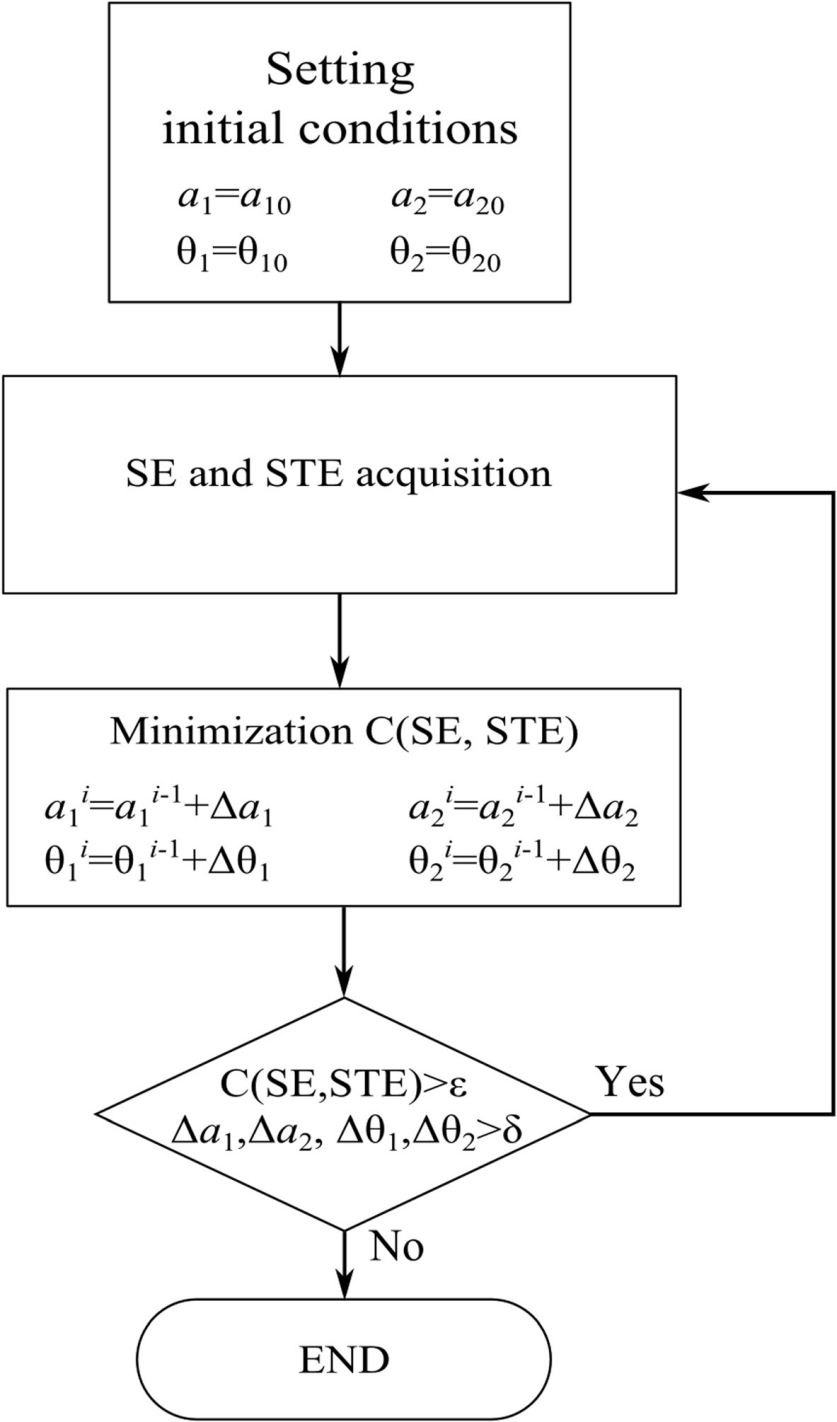
**The flow chart of the RF pulse calibration procedures.** The thresholds ε and δ are determined empirically.

Figure 3 shows the flow chart of the RF pulse calibration procedures. After acquiring the two echo signals at the *j*-th RF pulse sequence, the new shimming parameters are computed from the current echoes (**SE**_j_ and **STE**_j_) and the previous echoes (**SE**_j-1_, **STE**_j-1_) by minimizing the cost function. Then, the new shimming parameters are used to update the RF pulses to be applied at the next cycle. The acquisition of the echo signals and the computation of the shimming parameters are repeated until either the cost function value becomes smaller than the empirically determined thresholds.

The minimization has been performed by running the constrained nonlinear optimization algorithm, so called interior point algorithm. The minimization algorithm finds a solution with fewer iterations than other linear programing methods [18,19]. The built-in function in MATLAB (The Mathworks, Natick, USA), *fmincon*, has been used to implement the interior point algorithm.

**Figure 4:**
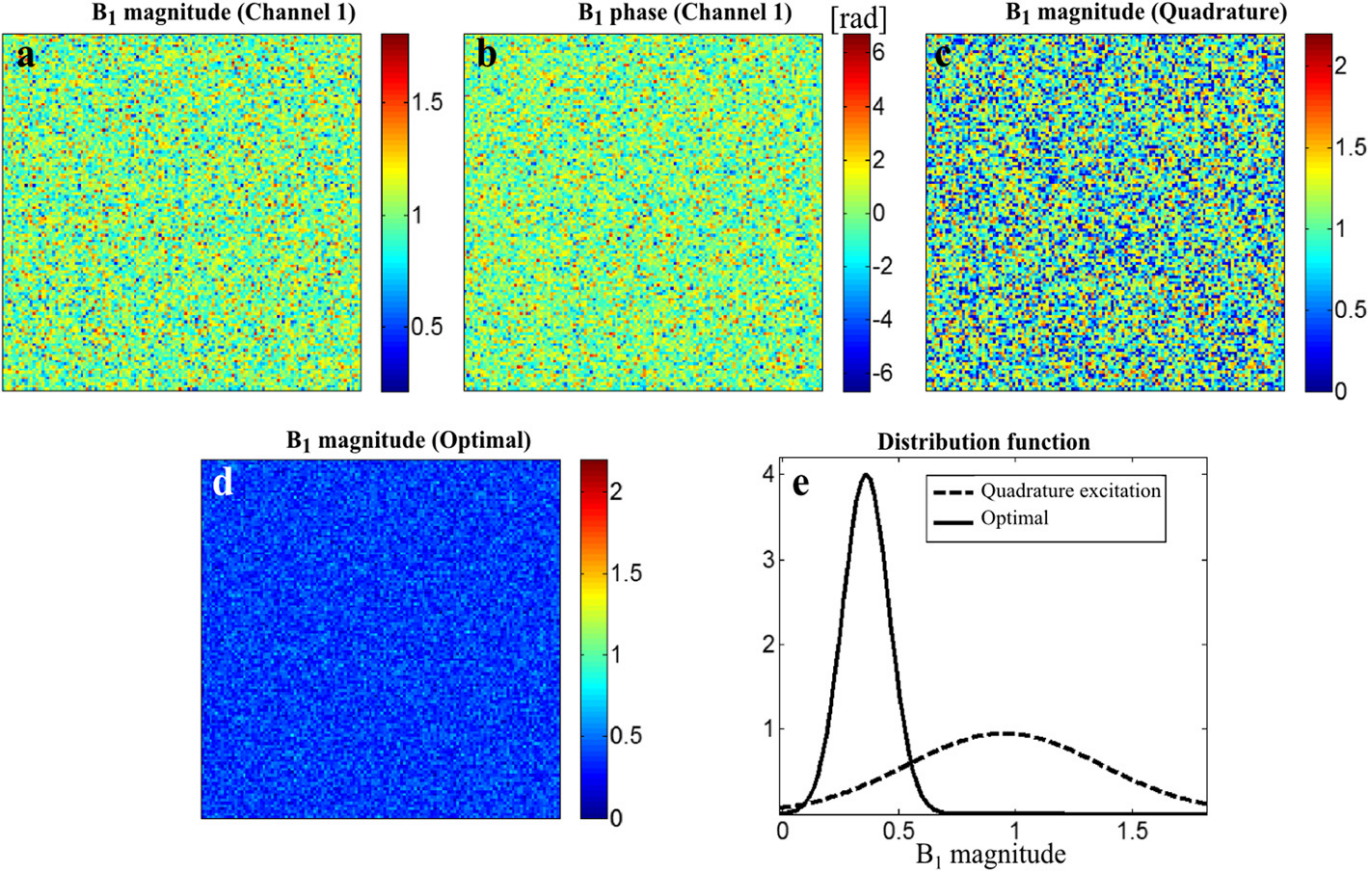
**The RF pulse calibration for the randomized B**_**1**_ **fields of the two-channel transmission system.** Each channel makes a random RF field in both magnitude and phase. **(a)** The magnitude and **(b)** phase distribution over (*x*, *y*) of the RF field generated by channel 1. **(c)** The composite field made by quadrature excitation. **(d)** The composite field made by the RF pulse calibration. **(e)** The magnitude distribution of the composite RF field generated by the quadrature excitation and the RF pulse calibration.

To demonstrate the **B**_1_ shimming tendency of the RF pulse calibration, two RF fields with random distribution in magnitude and phase have been generated over (*x*, *y*). Figure 4a and b show the magnitude and phase distribution of channel 1. The magnitude and phase distribution of channel 2 look similar to Figure 4a and b due to the randomness, so, they are not shown in the figure. The random fields have been considered as an extreme case of inhomogeneous field. Hence, the RF pulse calibration would result in improving the RF field homogeneity in the realistic situation if it improves the RF field homogeneity in this extreme case. The magnitude and phase have Gaussian distributions with the mean and standard deviation of (1, 0.2) and (0, π/2), respectively. If the two RF fields are combined in the quadrature mode, the magnitude of the composite field appears as shown in Figure 4c. If the optimization is applied to the two random RF fields, the composite field appears as shown in Figure 4d. The distribution of the magnitude of the composite field is shown in Figure 4e. The optimization has reduced the deviation from 0.42 to 0.09 demonstrating the **B**_1_ shimming tendency even in this extreme case of inhomogeneous field distribution.

### Verification of the RF pulse calibration using a FDTD model

To verify the **B**_1_ shimming tendency of the RF pulse calibration in the human body, the RF field generated by a high-pass birdcage coil in an adult human body model (Virtual family model, Duke [20]) has been computed. In the computation, a FDTD electromagnetic solver (SEMCAD X, Switzerland) has been used. The birdcage coil, with the diameter of 700 mm and the height of 820 mm, has 16 rungs and two excitation ports separated 90 degree apart. The birdcage coil has been tuned and matched at 123.5 MHz. The complex-numbered **B**_1_ maps have been taken from the torso region of the human model which consists of 35 different tissues with their own electrical conductivity and permittivity. The voxel size for the **B**_1_ maps was 3.57 × 3.57 × 5.46 mm^3^ on a FOV of 350 × 350 × 350 mm^3^ with a matrix size of 98 × 98 × 64. For the excitation of the birdcage coil at each port, a Gaussian pulse of the center frequency of 123.5 MHz and a bandwidth of 100 MHz was applied. The **B**_1_ map of one channel was computed with driving the corresponding channel with a voltage source and setting the other channel idle, and vice versa for the **B**_1_ map of the other channel. The two **B**_1_ maps have been combined in the quadrature mode as an initial condition for the optimization. The flip angle map was then computed from the composite field.

To compute the spin echo and stimulated echo signal using Eq. [9], the spin density attributed to each voxel of the FDTD human model was used in the integration. The transceiver phase *φ*(*x*, *y*) in Eq. [9] was assumed to be the phase of the composite field. To evaluate the flip angle uniformity after the optimization, the following figure of merit has been used,

10 $$ \eta =\sqrt{\frac{1}{N_X{N}_Y}{\displaystyle \sum_{i=1}^{N_X}{\displaystyle \sum_{j=1}^{N_Y}{\left|\frac{\alpha \left(i,j\right)-\overline{\alpha}\left(i,j\right)}{\overline{\alpha}\left(i,j\right)}\right|}^2}}}\times 100\left[\%\right] $$

in which (*i*, *j*) is the pixel index and (*N*_X_,*N*_Y_) is the number of pixels in the *x*- and *y*-direction.

### RF pulse calibration experiment at 3 T

RF pulse calibration experiments have been performed at a 3 T MRI system capable of two channel RF transmission. A head birdcage coil with the diameter of 290 mm and the height of 270 mm has been used for both transmission and reception at the imaging experiment of a water-filled phantom. The phantom consists of two identical cylinders with the diameter of 58 mm and the height of 180 mm. To make inhomogeneous RF field distribution in the phantom of a small size, asymmetric electrical conductivity has been made at the two bottles. One bottle was filled with 0.2 S/m NaCl solution with the other bottle filled with 1.8 S/m NaCl solution. The relative electrical permittivity of the solution was 81 for both bottles. The spin–lattice relaxation time of the solution at both bottles was about 500 ms.

The RF pulse in the calibration sequence was sinc-shaped with the pulse width of 2.6 ms and the bandwidth of 1.5 KHz. The timing *t*_1_ and *t*_2_ were 6 ms and 7 ms, respectively. The repetition time was 500 ms and the slice thickness was 10 mm. A MATLAB script function was made to acquire the spin and stimulated echo signals and to compute the next shimming parameters through the minimization. The next shimming parameters were used to update the driving RF pulse for each channel. The iteration was repeated until the minimization converged to the thresholds.

## Results and discussion

**Figure 5:**
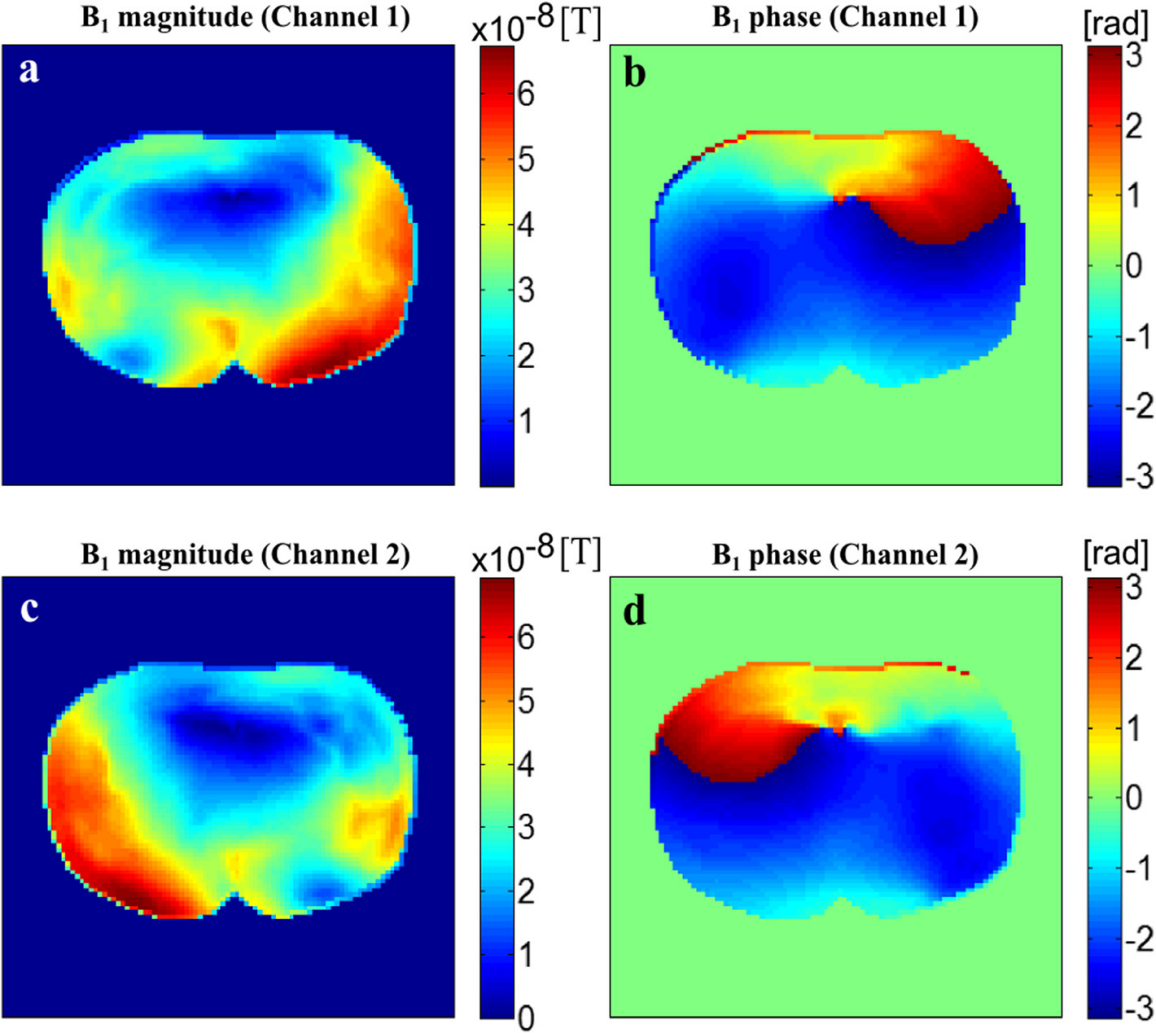
**The magnitude (a,c) and phase (b,d) maps of the B1 field generated by the birdcage coil.** The top **(a,b)** and bottom **(c,d)** rows. The top and bottom rows represent the channel 1 and channel 2 of the birdcage coil, respectively.

The **B**_1_ field map has been computed on a 3D matrix the size of 98 × 98 × 64 from the human body model placed in the two-port birdcage coil, and then, the RF pulse calibration has been made on a particular slice of interest. Figure 5 shows the magnitude and phase maps of the **B**_1_ fields of each channel on the middle axial plane of the human model. Both the magnitude and phase maps of each channel shows high inhomogeneity because of the attenuation and phase delay of the RF field in the electrically conductive human body with high electrical permittivity.

**Figure 6:**
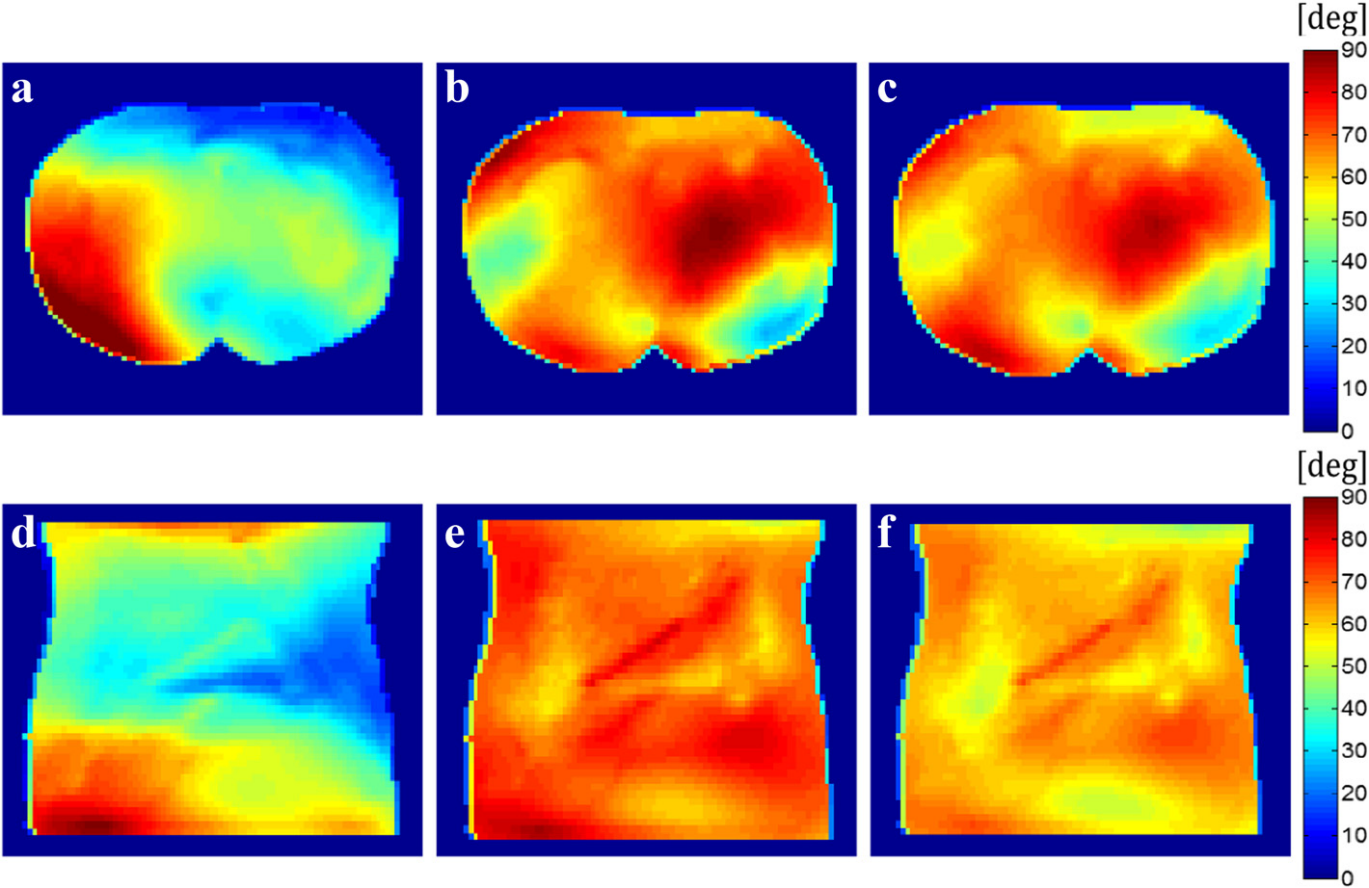
**Flip angle maps produced by the quadrature excitation (a,d), the iterative RF pulse calibration (b,e) and the B1 shimming (c,f).** The top **(a,b,c)** and bottom **(d,e,f)** rows. The top and bottom rows show the flip angle maps on an axial slice and a coronal slice, respectively. The RF pulse calibration has been made for each slice.

With the **B**_1_ field maps of each channel, the initial composite field has been made by combining the two fields in the quadrature mode. Figure 6a and d show the flip angle maps of the quadrature-mode fields on an axial slice and a coronal slice, respectively. The quadrature-mode fields have been used as an initial condition for the iterative RF pulse calibration. Figure 6b and e show the flip angle maps of the composite fields obtained by the iterative RF pulse calibration. The RF pulse calibration took 32 iterations on the axial slice and 31 iterations on the coronal slice. In Figure 6a, the uniformity of the flip angle is 27.4%. Whereas the uniformity of the flip angle is 13.5% in Figure 6b with the mean flip angle of 63.0°. The mean flip angle of 63.0° is near to the target value of 65.5°. The initial flip angle uniformity in Figure 6d is 18.0%, whereas the uniformity in Figure 6e is 8.0% with the mean flip angle of 68.0°. Figure 6c and f show the flip angle maps obtained after performing the conventional **B**_**1**_ shimming. In the conventional **B**_**1**_ shimming, **B**_**1**_ maps were first obtained for each channel, and then, the optimal magnitude and phase of the RF pulses were found to optimize the uniformity of the composite field. Table 1 summarizes the performance of the three methods which has been evaluated from the flip angle maps shown in Figure 6. In terms of the uniformity, the proposed method shows similar performance as compared to the **B**_**1**_ shimming method.

**Table 1:** **The B**_**1**_ **uniformity computed from the simulated flip angle maps and the magnitude ratio and phase difference in excitation**

	**Axial slice**			**Coronal slice**		
	**Quadrature excitation**	**RF pulse calibration**	**B**_**1**_ **shimming**	**Quadrature excitation**	**RF pulse calibration**	**B**_**1**_ **shimming**
η [%]	27.4	13.5	13.1	18.0	8.0	7.9
*a*_1_/*a*_2_	1.0	2.0	2.3	1.0	1.9	1.8
Δθ	90°	−50°	−40°	90°	−2°	−4°

Three methods, the quadrature excitation, the iterative RF pulse calibration and the **B**_1_ shimming, are compared for an axial and a coronal slice.

**Figure 7:**
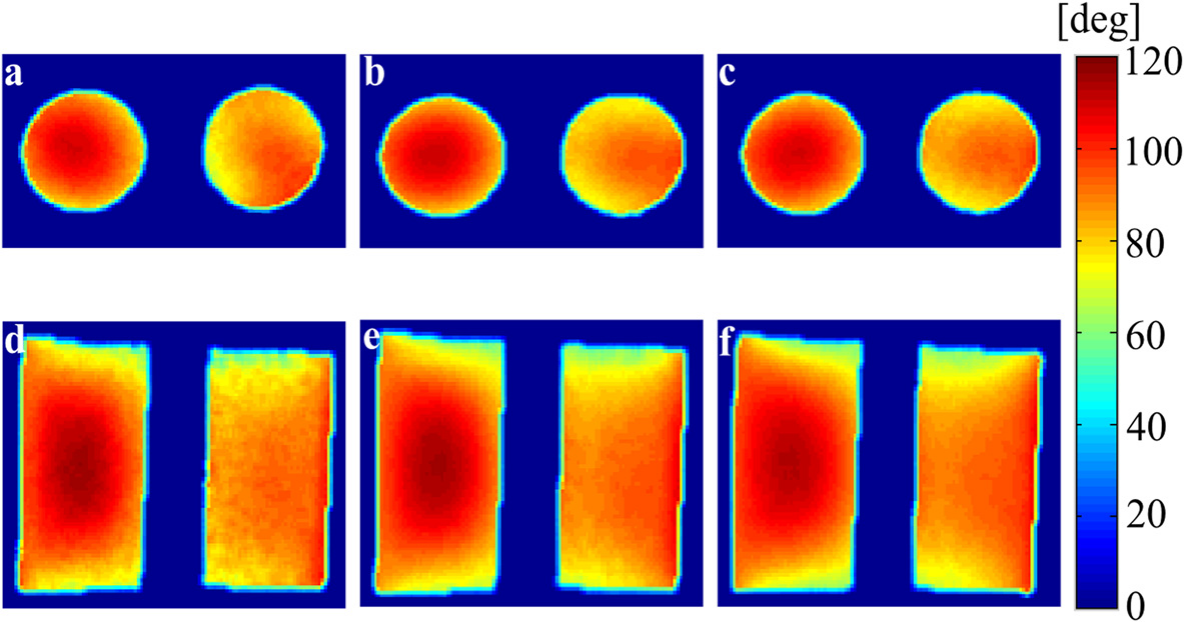
**Flip angle maps obtained by the double angle method (DAM).** The flip angle calibration for the nominal flip angle of 90 degrees has been done by the quadrature excitation **(a,d)**, the iterative RF pulse calibration **(b,e)** and the conventional B1 shimming **(c,f)** for an axial slice **(a,b,c)** and a coronal slice **(d,e,f)**.

Figure 7 shows the flip angle maps of the double-cylinder phantom when the nominal flip angles are set to 90°. The flip angle maps have been obtained with the double angle method (DAM) [6]. In the flip angle mapping with DAM, the TR/TE of the DAM sequence was 5000/18 ms with the slice thickness of 5 mm. The three methods, the quadrature-mode excitation, the iterative RF pulse calibration, and the **B**_**1**_ shimming, are compared in Figure 7. In the iterative RF pulse calibration, the slice thickness of the calibrating RF pulses was 10 mm and TR was 500 ms. In the RF pulse calibration of the axial and coronal slices, it took 36 and 31 iterations, respectively, to determine the optimal magnitudes and phases. The flip angle maps on an axial slice are shown in Figure 7a, b, c for the quadrature mode excitation, the iterative RF pulse calibration, and the **B**_**1**_ shimming, respectively. The flip angle maps on a coronal slice are also shown in Figure 7d, e, f, correspondingly. The mean flip angles on Figure 7b and e are 91.2° and 91.4°, respectively, demonstrating the flip angle calibration accuracy of the proposed method. Table 2 summarizes the uniformity of the flip angle maps shown in Figure 7 with the magnitude ratios and phase difference between the two channels. At the experiments too, the proposed method yielded similar **B**_**1**_ uniformity as compared to the **B**_**1**_ shimming method.

**Table 2:** **The B**_**1**_ **uniformity computed from the experimental flip angle maps and the magnitude ratio and phase difference in excitation**

	**Axial slice**			**Coronal slice**		
	**Quadrature excitation**	**RF pulse calibration**	**B**_**1**_ **shimming**	**Quadrature excitation**	**RF pulse calibration**	**B**_**1**_ **shimming**
η [%]	8.0	6.3	7.0	17.0	14.0	12.0
*a*_1_/*a*_2_	1.0	0.82	0.40	1.0	0.58	0.60
Δθ	90°	1.7°	−12°	90°	2°	−22°

Three methods, the quadrature excitation, the iterative RF pulse calibration and the B_1_ shimming, are compared for an axial and a coronal slice.

Since the RF pulse calibration is based on iterative search of the optimal driving voltages, the convergence may vary scan by scan depending on the noise and RF system instability. Table 3 shows an example of the number of iterations at the RF pulse calibration on a certain slice of the double-cylinder phantom. In ten trials of the RF pulse calibration with TR of 500 ms and slice thickness of 10 mm, the number of iterations varied from 34 to 57 with small variance in the magnitude ratio, phase difference, and **B**_**1**_ uniformity. The follow-up gradient echo imaging scan also showed little difference in image uniformity despite the variance of the magnitude ratio and phase difference. In the FDTD simulation, it has been observed that the iterative search of the optimal voltages may fail if the noise level is too high. With SNR of the spin echo and stimulated echo signals higher than 28 dB, the RF pulse calibration converged within a few tens of iterations.

**Table 3:** **RF pulse calibration output at 10 trials of the experiment**

	**Initial condition**	**RF pulse calibration output**									
		**Trial #1**	**Trial #2**	**Trial #3**	**Trial #4**	**Trial #5**	**Trial #6**	**Trial #7**	**Trial #8**	**Trial #9**	**Trial #10**
No. of iterations		43	40	43	57	36	34	35	34	38	35
*a*_*1*_ */a*_*2*_	1	0.74	0.73	0.74	0.74	0.74	0.70	0.70	0.71	0.71	0.71
Δθ [deg]	90	91.3	92.2	90.6	90.4	90.7	90.8	91	90.7	90.7	90.9
η [%]	21.44	15.0	15.2	14.7	14.6	14.9	14.9	15.0	14.9	14.9	14.9

The iterative RF pulse calibration used the quadrature-mode excitation as an initial condition.

Due to some difficulties in spectrometer programming, the RF pulse calibration experiments have been performed ignoring the T_1_ effects. That is, after setting the new RF voltages for the next cycle of iteration, the spin and stimulated echoes were acquired after repeating a few RF pulse sequences to saturate the T_1_ effects. If the spin and stimulated echoes are acquired at the very next cycle of the RF pulse sequence with short TR, the amplitude of the spin and stimulated echoes will be affected by the T_1_ effects as well as the RF voltage changes. The T_1_ effects on the RF pulse calibration should be investigated to use the RF pulse calibration with short TR. Since the computation time for one iteration is an order of ms, the computation time would not be a hurdle to implement the RF pulse calibration in a short TR configuration.

Since the number of iterations is an order of a few tens in most cases, the number of pulse repetitions in the RF pulse calibration is much smaller than is required in **B**_1_ mapping. In **B**_1_ mapping, a number of phase encodings are necessary for each channel and for magnitude and phase mapping, respectively. Therefore, the scan time for the RF pulse calibration would be much shorter than is for the **B**_1_ mapping as long as similar TRs are employed in both cases. The present study is limited to two-channel excitation, but, the general principle of the present study can be applied to multi-channel excitation with the number of channels higher than two. In ultra-high field MRI with the main field strength higher than 7 T, eight-channel RF transmission or more is now used to overcome the **B**_1_ inhomogeneity [21,22]. As the number of transmission channels increases for more efficient **B**_1_ shimming, **B**_1_ mapping time for every channel also increases. Therefore, long **B**_1_ mapping time may become a big hurdle in applying **B**_1_ shimming to clinical studies. Although many fast **B**_1_ mapping techniques have been proposed to reduce the scan time of multi-channel **B**_1_ mapping, implementing the multi-channel **B**_1_ mapping in the pre-scan frame would be troublesome. It is expected that the proposed method can be also used for **B**_1_ shimming in high field MRI with the number of transmission channels higher than two. The proposed method has a limitation that **B**_1_ shimming at a region of interest (ROI) inside the entire slice is not possible since the spin echo and stimulated echo signals are acquired from the entire slice. In contrast, the conventional **B**_1_ shimming can be applied to a small region of interest by taking the fields only at the ROI into consideration for the optimization.

## Conclusions

A new method for fast RF pulse calibration with **B**_1_ shimming capability has been proposed. Since the proposed RF pulse calibration is not based on time-consuming **B**_1_ mapping, the RF pulse calibration time could be much shorter than the **B**_1_-mapping based methods. The proposed method is expected to be a practical substitute for the **B**_1_-mapping based **B**_1_ shimming methods when long pre-scan time is not tolerable.
